# Supplementary material for: Targeting ADAR stability: a novel approach to combat glioblastoma
Source: Front Oncol. 2026 Aug 6;16:1827793. doi: 10.3389/fonc.2026.1827793 (PMC13489870; doi:10.3389/fonc.2026.1827793)
Supplement: Supplementary file 1 [file SupplementaryFile1.zip › Supplementary file 1/Glycosphingolipid_biosynthesis_lacto_and_neolacto_series.pdf]

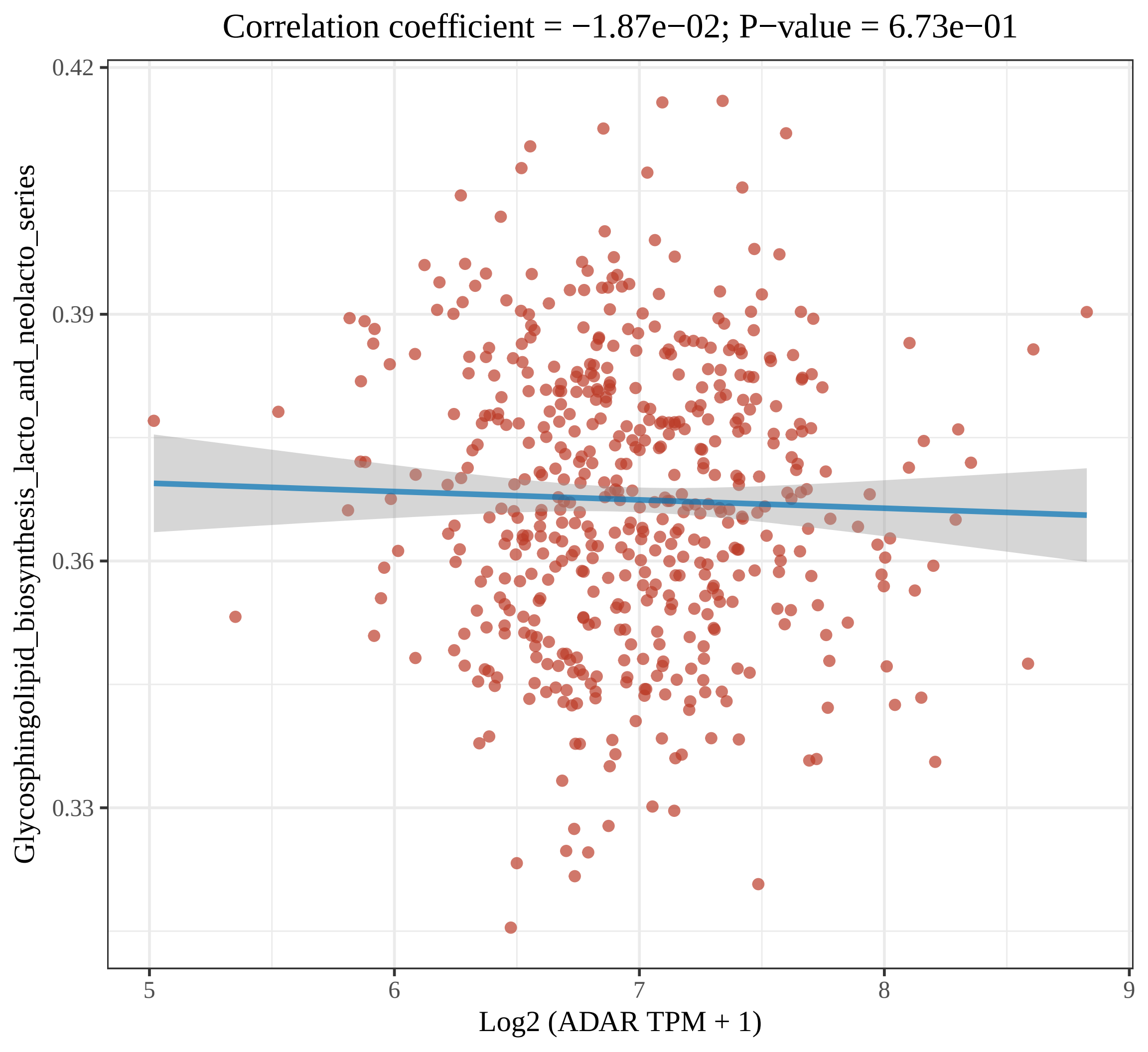
Correlation coefficient = −1.87e−02; P−value = 6.73e−01
Glycosphingolipid_biosynthesis_lacto_and_neolacto_series
0.42
0.39
0.36
0.33
5
6
7
8
9
Log2 (ADAR TPM + 1)
